# On-Surface Synthesis of Azapolyarenes and Their Bromine-Assisted Chiral Self-Assembly

**DOI:** 10.1021/acsami.6c07305

**Published:** 2026-05-01

**Authors:** Yi Zhang, Jianchen Lu, Wuyi Gao, Yong Zhang, Lei Gao, Karl-Heinz Ernst, Jinming Cai

**Affiliations:** † Faculty of Materials Science and Engineering, 47910 Kunming University of Science and Technology, 68 Wenchang Road, Kunming 650093, China; ‡ Southwest United Graduate School, 298, 121 Street, Kunming 650092, China; § Faculty of Science, Kunming University of Science and Technology, 727 Jingming South Road, Kunming 650500, China; ∥ Empa, Swiss Federal Laboratories for Materials Science and Technology, Überlandstrasse 129, Dübendorf 8600, Switzerland; ⊥ Nanosurf Laboratory, Institute of Physics, The Czech Academy of Sciences, Cukrovarnická 10, Prague 16200, Czech Republic; # Department of Chemistry, University of Zurich, Winterthurerstrasse 190, Zürich 8057, Switzerland

**Keywords:** on-surface synthesis, STM, chirality, Ullmann C−C coupling, C−N cross-coupling

## Abstract

The rational design of molecular precursors that direct divergent surface reactions offers powerful opportunities for controlling the formation and organization of heteroatom-doped π-conjugated nanostructures. Here, we report the on-surface synthesis of three distinct azapolyaromatic compounds—a helicene, a pyrazine, and a nanographene—obtained from a single brominated precursor on Au(111). Thermal activation triggers selective dehalogenation, C–C or C–N coupling, and intramolecular cyclodehydrogenation, giving rise to structurally defined products whose structures were elucidated by scanning tunneling microscopy and density functional theory calculations. Remarkably, the resulting species undergo bromine-assisted self-assembly into phase-separated two-dimensional domains, reflecting differences in molecular chirality, planarity, and intermolecular interactions. These findings highlight the potential of programmed on-surface chemistry for generating multiple heteroatom-doped aromatic architectures from a single feedstock, providing a versatile strategy for the atomically precise construction of functional nanographenes and chiral π-systems.

## Graphical abstract

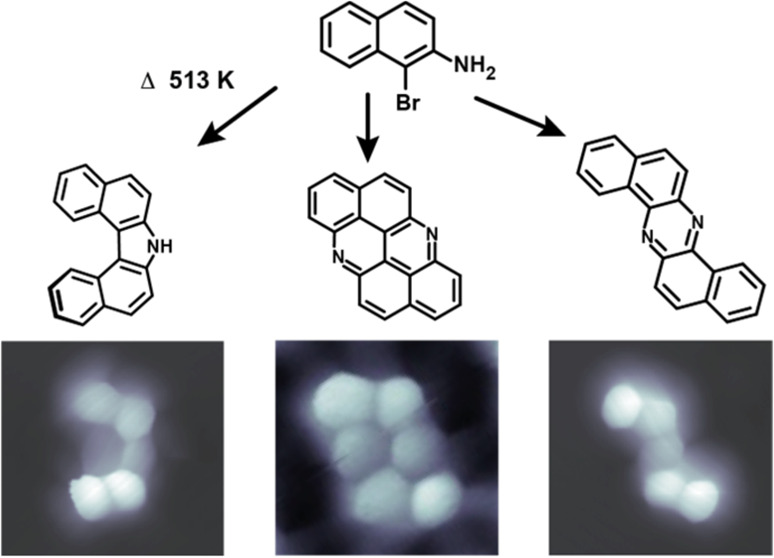

## 1 Introduction

The atomically precise construction of polycyclic aromatic hydrocarbons (PAHs) and molecular nanographenes lies at the heart of efforts to design functional carbon-based materials.^1,2^ While traditional solution-phase strategies have yielded remarkable π-conjugated systems, they often reach intrinsic limits as molecular complexity grows or as atomic-scale precision becomes essential. Anthanthrene, composed of two laterally fused anthracene units, exemplifies these challenges: despite its appealing electronic structure and promise in organic electronics,^3–5^ its synthesis still relies on only a few established routes.^6^

Helicenes, another key class of ortho-fused PAHs, combine structural rigidity, extended conjugation, and inherent chirality,^7,8^ making them ideal systems for exploring two-dimensional chiral self-assembly,^9^ on-surface chemistry,^10^ and the chirality-induced spin selectivity (CISS) effect.^11–14^

A central frontier in this field involves the introduction of heteroatoms, particularly nitrogen, into carbon-rich scaffolds to modulate electronic structure, local reactivity, and intermolecular interactions. Especially, nitrogen-doped nanographenes exhibit tailored band gaps, and enhanced charge transport.^15,16^

Bridging such different PAH systems with scalable synthetic control remains a formidable goal toward new devices. To overcome these challenges, the strategy of on-surface synthesis (OSS) has emerged as a powerful bottom-up approach for the fabrication of atomically precise nanostructures.^17,18^ By confining reactions to two dimensions, OSS enables the generation of otherwise inaccessible products, including nanographenes,^19^ porous covalent networks,^20^ graphene nanoribbons,^21^ and heteroatom-doped conjugated frameworks.^22^

Here we report OSS of different nitrogen containing PAHs from a single rationally designed chemical precursor on a gold(111) surface (Scheme 1). Products are characterized and identified by scanning tunneling microscopy (STM) and bond-resolved STM (BR-STM) with a single carbon monoxide molecule at the tip, supported by density functional theory (DFT). Three products were identified, namely, so-called Meisenheimer helicene (1,1′-binaphthyl-2,2′-imine, **1**), 6,12-diazaanthanthrene (acridino­[2,1,9,8-klmna]­acridine, **2**), and dibenzo­[*a*,*h*]­phenazine (dinaphtho­[2,3-b:2′,3′-*f*]­pyrazine, **3**) (Scheme 1). Released by Ullmann coupling, bromine atoms mediate two-dimensional self-assembly of the three products into phase-separated domains, meaning that no mixing of different species into same ordered domains is observed. As all surface-bound PAH-products are chiral, subtle differences in respect of chiral assembly are observed.

**Scheme 1:**
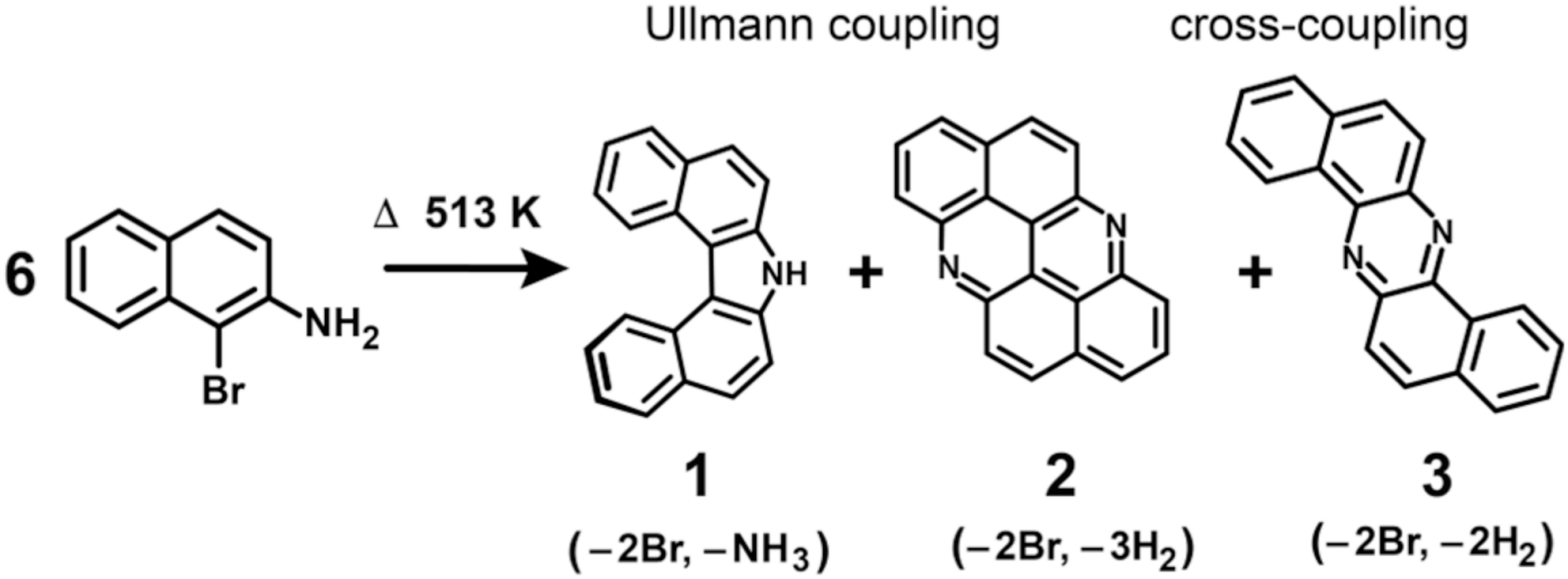
On-Surface Synthesis Pathways to Meisenheimer Helicene (1), 6,12-Diazaanthanthrene (2) and Dibenzo­[*a*,*h*]­phenazine (3)

## 2 Methods Section

### 2.1 Experimental Details

STM measurements were performed with a commercial low-temperature STM from Scienta Omicron operating at 4.2 K and base pressures below 1 × 10^–10^ mbar. The Au(111) single crystal was cleaned by repeated Ar^+^ sputtering and annealing cycles of 650 K. Sample annealing temperature was controlled via a K-type thermocouple directly spot-welded onto the crystal. The precursor (Bide pharmatech Co., Ltd.) molecules were evaporated from a Knudsen-cell style molecular evaporator held at RT. All STM images were acquired in constant-current mode. BR-STM measurements were performed with a tungsten tip in constant-height mode. The tip was functionalized by a controlled pickup of a single adsorbed CO molecule to the tip apex. All STM images were processed with WSxM software.

### 2.2 Theoretical Details

All density functional theory (DFT) calculations were conducted using the Vienna Ab initio Simulation Package (VASP) with the Perdew–Burke–Ernzerhof (PBE) generalized gradient approximation for the exchange–correlation functional. The interactions between the core and valence electrons were treated with the projector augmented-wave (PAW) method. A kinetic energy cutoff of 600 eV was applied for the plane-wave basis set. The Au(111) surface was modeled using a three-layer slab, with the bottom two layers fixed at their bulk positions and the top layer fully relaxed. This approach has been tested for robustness for product **1** by performing calculations also for 4-, 5- and 6-layer-models, leading to basically identical adsorbate structures (Figure S1)

Structural optimizations were carried out until the residual atomic forces were smaller than 0.02 eV/Å and the total energy variation was below 10^–4^ eV. Dispersion forces were accounted for through the DFT-D3 correction. A vacuum spacing exceeding 20 Å was added along the out-of-plane direction to suppress interlayer interactions. Geometry optimizations were performed with Γ-point-only sampling of the first Brillouin zone. The transition-state searches were performed using the climbing-image nudged elastic band (CI-NEB) method to determine the minimum energy pathways (MEPs) and activation energies. All intermediate images were relaxed until the maximum force perpendicular to the reaction coordinate was below 0.05 eV/Å.

## 3 Results and Discussion

Upon deposition of 2-amino-1-bromonaphthalene on Au(111) held at 513 K, the three species shown in Scheme 1 self-assemble into compositionally pure, separate islands without intermolecular mixing (Figure 1a,b). Notably, the characteristic herringbone reconstruction of Au(111) is retained only in the uncovered surface regions between molecular domains. All molecular islands exhibit bright circular protrusions in the STM images, assigned to bromine atoms that were cleaved from the precursor during Ullmann-type C–C coupling reactions.

**Figure 1:**
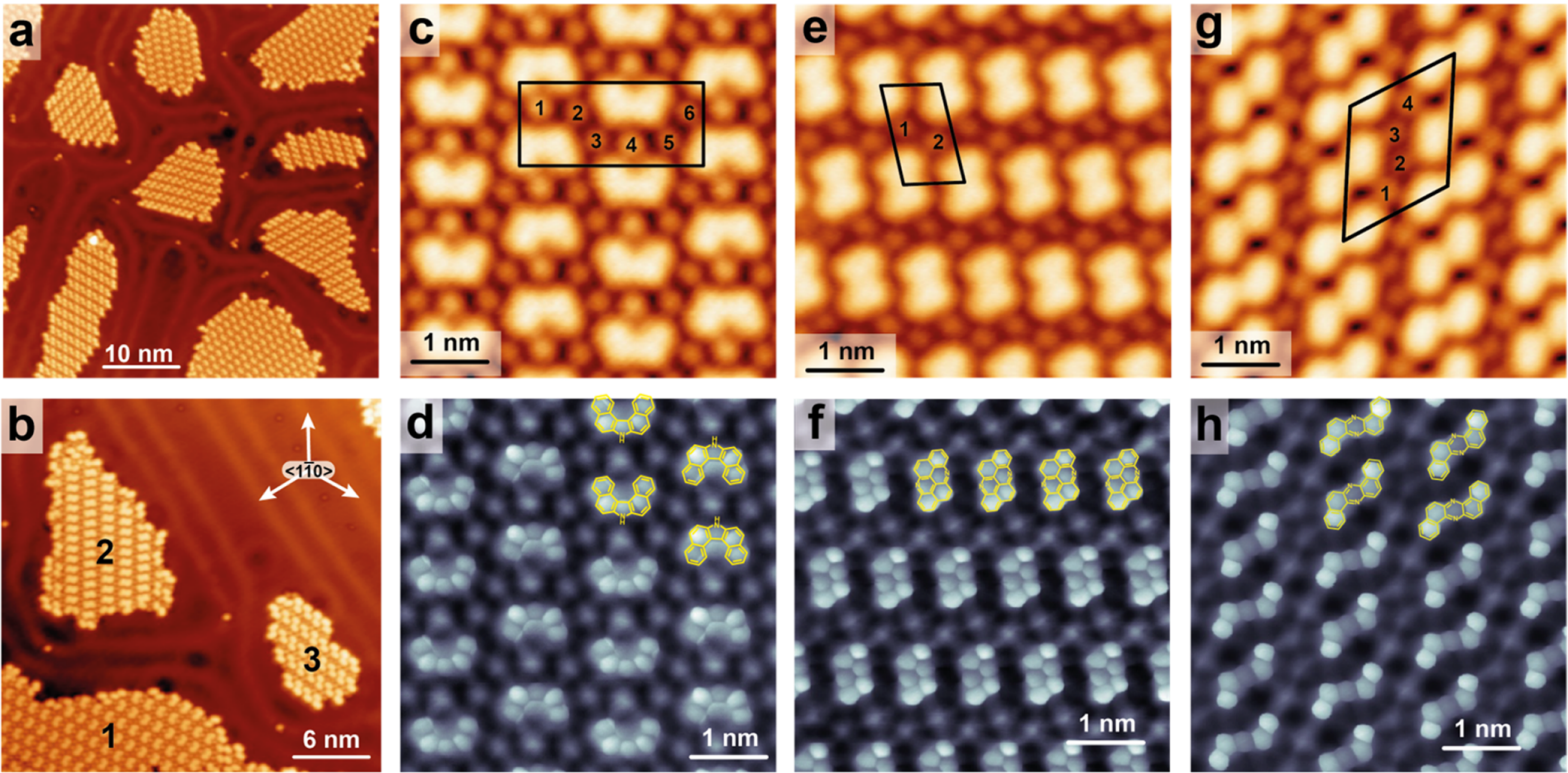
STM images of dimeric on-surface coupling products of 2-amino-1-bromonaphthalene on Au(111). (a) Overview STM image showing molecular islands. (b) STM image showing islands of three different products, labeled according to Scheme 1. (c) STM image of part of an island composed of **1** embedded between bromine atoms. The 2D unit cell is indicated as rectangle. Six bromine atoms per unit cell are labeled. (d) BR-STM image of area shown in (c). (e) STM image of part of an island composed of **2** and bromine atoms. The adlayer unit cell is indicated as parallelogram, with 2 bromine atoms per unit cell. (f) BR-STM image of area shown in (e). (g) STM image of part of an island composed of **3** plus bromine atoms. The adlayer unit cell is indicated as parallelogram, with 4 bromine atoms per unit cell. (h) BR-STM image of area shown in (g) The chemical models corresponding to the three products are superimposed in figures d, f, and h, respectively. Scanning parameters: (a, b) *U* = 50 mV, I = 50 pA; (c, e, g) *U* = 50 mV, *I* = 120 pA. (d, f, h) *U* = 2 mV, *I* = 50 pA. The surface directions indicated in (b) apply to all STM images shown.

Product **1** is identified as 1,1′-binaphthyl-2,2′-imine (Figure 1c,d), a compound representing the first synthetically prepared helical polyarene (**1** in Scheme 1). Although this molecule is commonly attributed to Jakob Meisenheimer,^23,24^ it was in fact first reported in 1882 by Hans Walder from the laboratory of Victor Merz at the University of Zurich.^25^ After an initial Ullmann C–C coupling, the *syn*-configured intermediate undergoes thermally induced NH_3_ elimination, yielding the helical imine structure (Scheme S1).

STM images of product **2** (Figure 1e,f) reveal an anthanthrene-like backbone. Its formation also begins with an Ullmann coupling, but when the intermediate adopts an *anti*-configuration, subsequent cyclodehydrogenation yields diazaanthanthrene, with nitrogen atoms incorporated at the 6,12-positions (Scheme S1). Note that analogues of **1** and **2**, with N (or NH) substituted by oxygen, have been observed in OSS when starting with brominated 1,1′-bi-2-naphthol.^26^

Finally, product **3** (Figures 1g,h) is assigned as dibenzo­[*a*,*h*]­phenazine, formed through a C–N cross-coupling following precursor dehydro-bromination (Scheme S1). For the detailed reaction mechanisms it is expected that gold adatoms are involved. For on-surface dehydrogenation, for example, it has been shown that such scenario results in much lower activation barriers.^27,28^ STM images after annealing to 433 K suggest organo-metallic intermediates, while DFT calculations show that including a gold adatom indeed results in lower energy of one intermediate in the reaction path to product **3** (Figure S2).

All three products coself-assemble with bromine into their respective molecular domains, a behavior commonly observed following on-surface Ullmann coupling.^29^ Although dehydrogenation also occurs, bromine remains on the surface. Previous studies have shown that, under comparable conditions on Au(111), dehydrogenation of PAHs results in HBr desorption.^30^ The continued presence of bromine in the present case therefore points to alternative decomposition pathways, likely dominated by debromination rather than dehydrogenation.

Species **3** dominates under the chosen conditions (*T*_Sample_ = 513 K). Out of 1080 molecules counted, 713 (66%) correspond to product **3**, 313 (29%) to product **1**, and 54 (5%) to product **2**. In contrast, when deposition is carried out during slow annealing from room temperature to 514 K, product 3 becomes even more prevalent, accounting for 82% of the observed molecules, followed by product **1** (17%), while product **2** is rarely detected (≈1%). A similar distribution is obtained when deposition is performed at room temperature followed by stepwise annealing, first to 433 K and then, after a 1 min hold, further to 473 K. The product ratio shifts even more dramatically when the substrate is held at 553 K, under which conditions products **1** and **2** are scarcely observed.

Although products **2** and **3** are planar, both act as chiral adsorbates and form homochiral 2D domains (Figure 1e–h). For product **2**, however, rows of the other enantiomer can occasionally be incorporated (Figure S3). Such observation suggests that the energetic difference between homo- and heterochiral arrangements is small. The unit cell for domains of **2** (Figure 1e) contains one molecule and two bromine atoms, consistent with the reaction stoichiometry, as two bromine atoms are released during the coupling. In the domains of product **3**, adjacent molecules within the rows adopt slightly different orientations. Consequently, the unit cell of the self-assembled domains of **3** contains two molecules and four bromine atoms (Figure 1g), in agreement with the reaction stoichiometry. In contrast, the unit cell of product **1** contains two molecules and six bromine atoms, indicating an excess of bromine likely arising from side reactions. As a consequence, areas with highly disordered debris are also observed (Figure S4). An excess of bromine associated with homochiral self-assembly has also been reported in on-surface Ullmann coupling to trishelicenes.^31^

Analysis of the chiral self-assembly of product **1** requires sufficient height resolution in STM (Figure S5). BR-STM images of individual molecules show that the naphthyl group on one side is indeed elevated, as highlighted by the distal benzo group marked in yellow in Figure 2a. BR-STM imaging of a molecular island of **1** reveals heterochiral self-assembly (Figure 2b). These results demonstrate that despite surface confinement the strained helical architecture is retained while simultaneously dictating enantiomeric organization. Such behavior is reminiscent of the (bromine-free) self-assembly of higher helicenes, as observed for heptahelicene, for example, on the same surface.^32^ DFT modeling of the isolated adsorbate confirms its nonplanar geometry (Figure 2c,d), which arises from steric repulsion between the two inner hydrogen atoms. To evaluate structural changes from the gas phase to the adsorbed state on Au(111), the dihedral angle between the two terminal benzo groups was calculated for the free molecule, an isolated adsorbate, and the adsorbate within the self-assembled domain. The dihedral angle decreases significantly upon adsorption, while self-assembly induces only minor additional changes (Figure 3). This indicates that interactions with the substrate have a dominant influence on the molecular geometry, whereas lateral interactions with bromine induce only minor changes in the dihedral angle.

**Figure 2:**
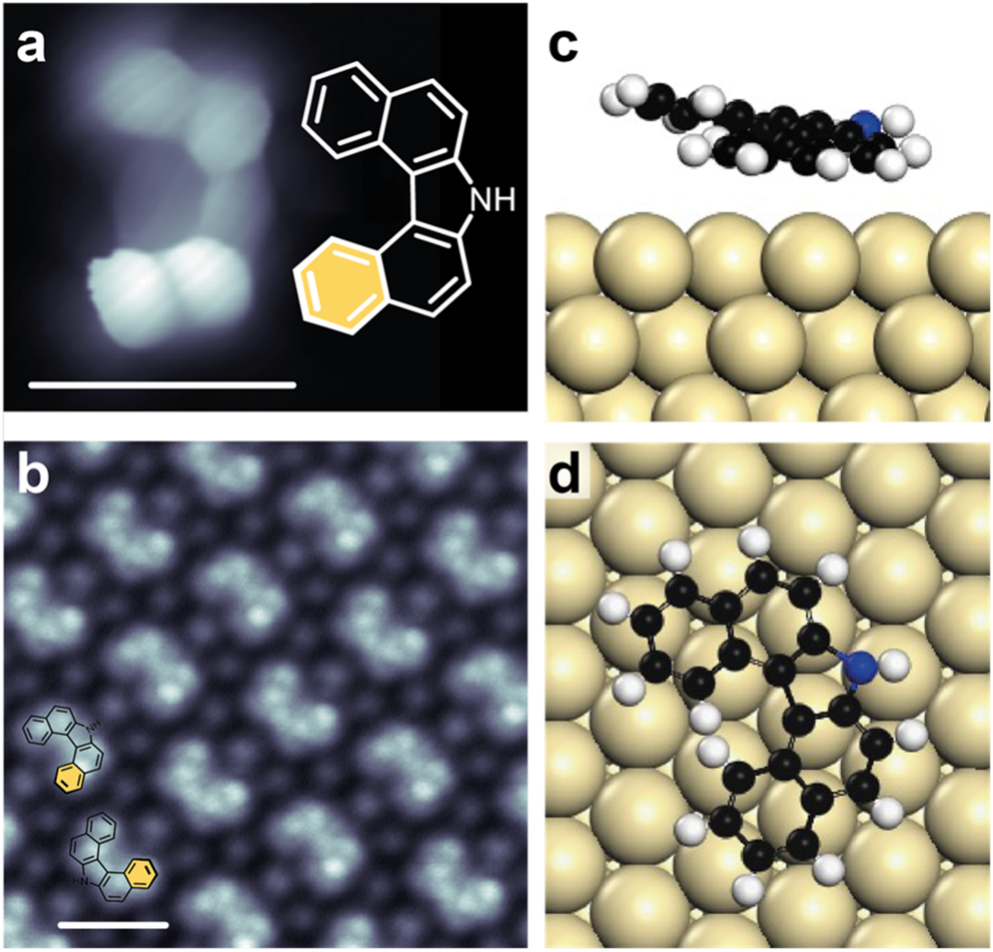
Self-assembly and nonplanarity of **1**. (a) BR-STM image of a single helicene and a sketch of **1** indicating the distal ring in yellow. (b) BR-STM image of a domain of **1**. The helicene forms homochiral rows but heterochiral domains. (scale bars in a and b are 1 nm) (c) Side view of calculated adsorbate complex. (d) Top view of calculated adsorbate complex. Scanning parameters: (a, b) *U* = 2 mV, *I* = 50 pA.

**Figure 3:**
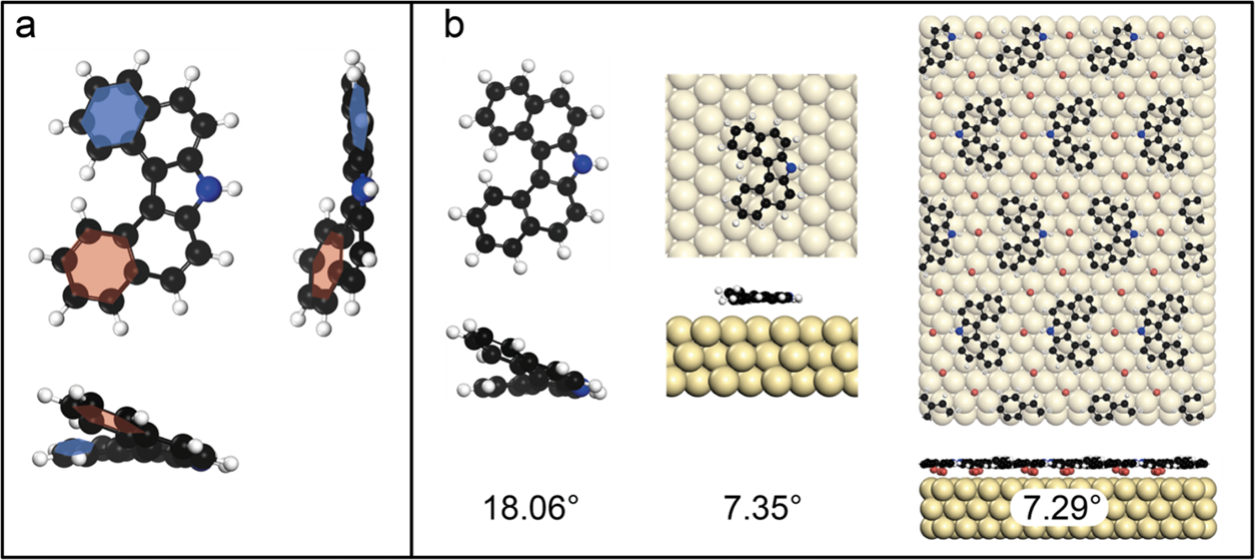
DFT calculations of the helicity of **1**. (a) The helicity was quantified as the dihedral angle between the two terminal benzo groups (indicated by the blue and red planes). (b) From the free molecule (18.06°) the dihedral angle decreases substantially to 7.35° on the surface. Coadsorbed bromine has no significant effect.

For chiral self-assembly, the barrier of interconversion between the enantiomers is of interest. Using climbing-image nudged elastic band (CI-NEB) calculations the minimum energy pathway and corresponding activation energies were evaluated. The resulting barrier for enantiomerization of **1** on the surface is remarkably low (Figure 4). Assuming a symmetric, planar transition state, we calculate a barrier of 46 meV. By comparison, the racemization barrier of free [4]­helicene ranges—depending on the density functional employed—from 36 to 46 meV,^33,34^ whereas higher homologues of all-carbon [*n*]­helicenes (*n* = 7–9) exhibit racemization energies of around 425 meV.^35^

**Figure 4:**
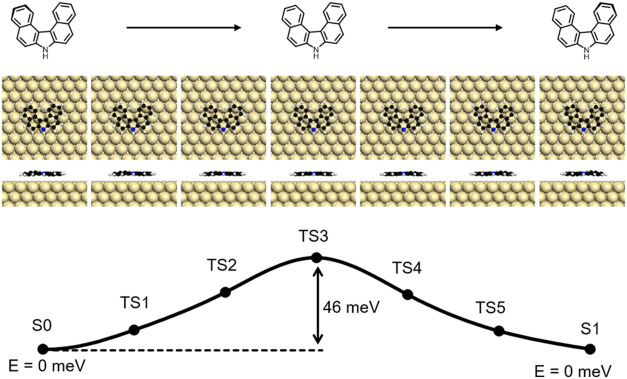
DFT energy calculation of several transition states during enantio-conversion from *(M)-* to *(P)-*helicene.

In contrast to **1**, domains of **3** are homochiral. A DFT evaluation of homochiral versus heterochiral self-assembly for **1** and **3**, performed both in the presence and absence of bromine atoms, is broadly consistent with the experimental observations. In general, structures containing bromine are substantially lower in energy (Figure S6). The calculations favor heterochiral assembly for **1** and homochiral domains for **3**; however, the associated energy differences are relatively small (Table 1).

**Table 1:** Relative Energies (Per Unit Cell Containing Two Molecules) of Chiral Self-Assembly for Products **1** and **3^a^**

	with Br		without Br	
	homo	hetero	homo	hetero
**1**	+20 meV	0	+30 meV	0
**3**	0	+100 meV	0	+30 meV

a Low-energy assembly is set to zero eV.

## 4 Conclusions

In summary, we show that a single brominated amine precursor can yield three distinct nitrogen-containing polycyclic aromatic hydrocarbons on Au(111) *via* selective on-surface reactions. Controlled C–C and C–N coupling, together with cyclodehydrogenation, produces a helicene, a diazananographene, and a phenazine analogue, as identified by bond-resolved STM. Bromine released during the reactions mediates self-assembly into phase-separated, compositionally pure domains that reflect differences in molecular chirality and planarity. These results underscore the power of on-surface synthesis to generate multiple heteroatom-doped π-conjugated architectures from a single molecular feedstock. In this context, further derivatization at both ends of the precursor could provide a pathway toward nitrogen-containing polyaromatic nanoribbons.

## Supplementary Material



## Notes

DFT calculations for different layer-slabs and of homo- versus heterochiral assembly, tentative reaction paths and STM images of 2D self-assembled domains, of ordered and disordered regions, as well as a BR-STM image from a domain of **1** at different scan heights (PDF)
