# Supplementary material for: On-Surface Synthesis of Azapolyarenes and Their Bromine-Assisted Chiral Self-Assembly
Source: ACS Appl Mater Interfaces. 2026 May 1;18(18):27109–14. doi: 10.1021/acsami.6c07305 (PMC13235640; doi:10.1021/acsami.6c07305)
Supplement: Supplementary file 1 [file am6c07305_si_001.pdf]

Supporting Information

On-surface synthesis of azapolyarenes and their bromine-assisted chiral self-assembly

Yi Zhang,[†,∥,#] Jianchen Lu,[†,#] Wuyi Gao,[†] Yong Zhang,[†] Lei Gao,[¶] Karl-Heinz Ernst,[§,‡,¥,*] and Jinming Cai[†,∥,*]

[†] Faculty of Materials Science and Engineering, Kunming University of Science and Technology, 68 Wenchang Road, Kunming 650093, China

[∥] Southwest United Graduate School, 298, 121 Street, Kunming, Yunnan 650093, China

[¶] Faculty of Science, Kunming University of Science and Technology, 727 Jingming South Road, Kunming 650500, China

[‡] Empa, Swiss Federal Laboratories for Materials Science and Technology, 8600 Dübendorf, Switzerland

[§] Nanosurf Laboratory, Institute of Physics, The Czech Academy of Sciences, 16200 Prague, Czech Republic

[¥] Department of Chemistry, University of Zürich, 8057 Zürich, Switzerland

e-mail: kalle@fzu.cz; j.cai@kust.edu.cn

[#] These authors contributed equally.

Content

1. Comparison of 3-to-6-layer-DFT calculations for **1** 2

2. Tentative reaction paths 2, 3

3. STM images of 2D self-assembled domains 3

4. STM images containing ordered and disordered regions. 4

5. BR-STM images of **1** at different scan heights 4

6. DFT calculations of homo- *versus* heterochiral assembly for **1** and **3** 5

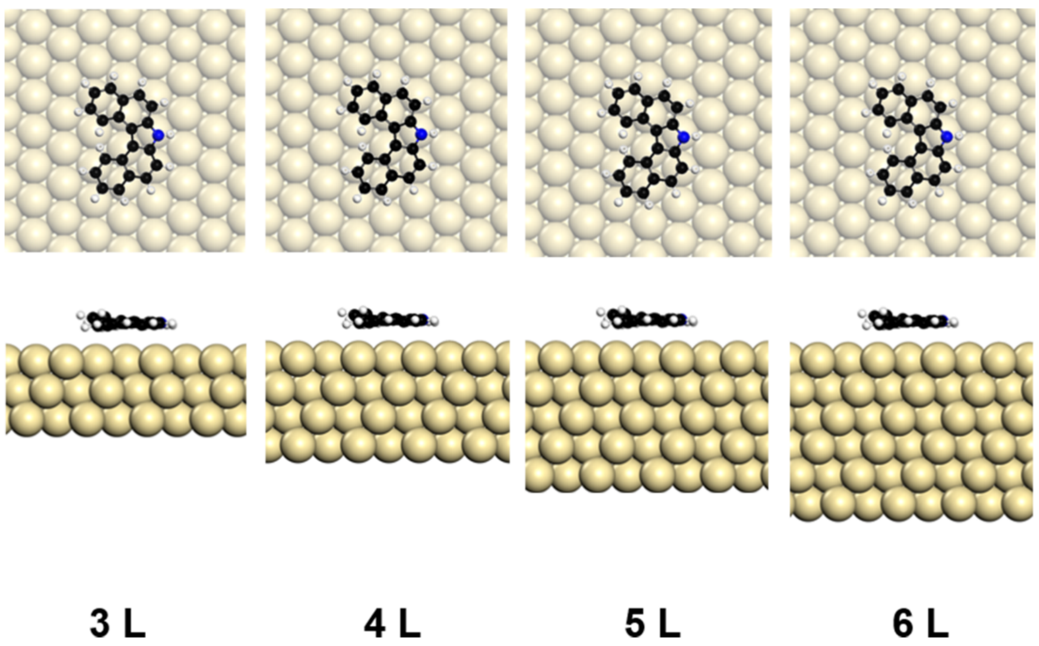

Figure S1. Effect of slab thickness on the molecular structure. Optimized structures of the adsorbed molecule on Au(111) with 3, 4, 5, and 6 layers (denoted as 3L-6L, respectively) are basically identical.

Scheme S1. Tentative reaction schemes for the observed products. Ammonia and hydrogen, after recombination to molecular hydrogen, desorb from the surface.

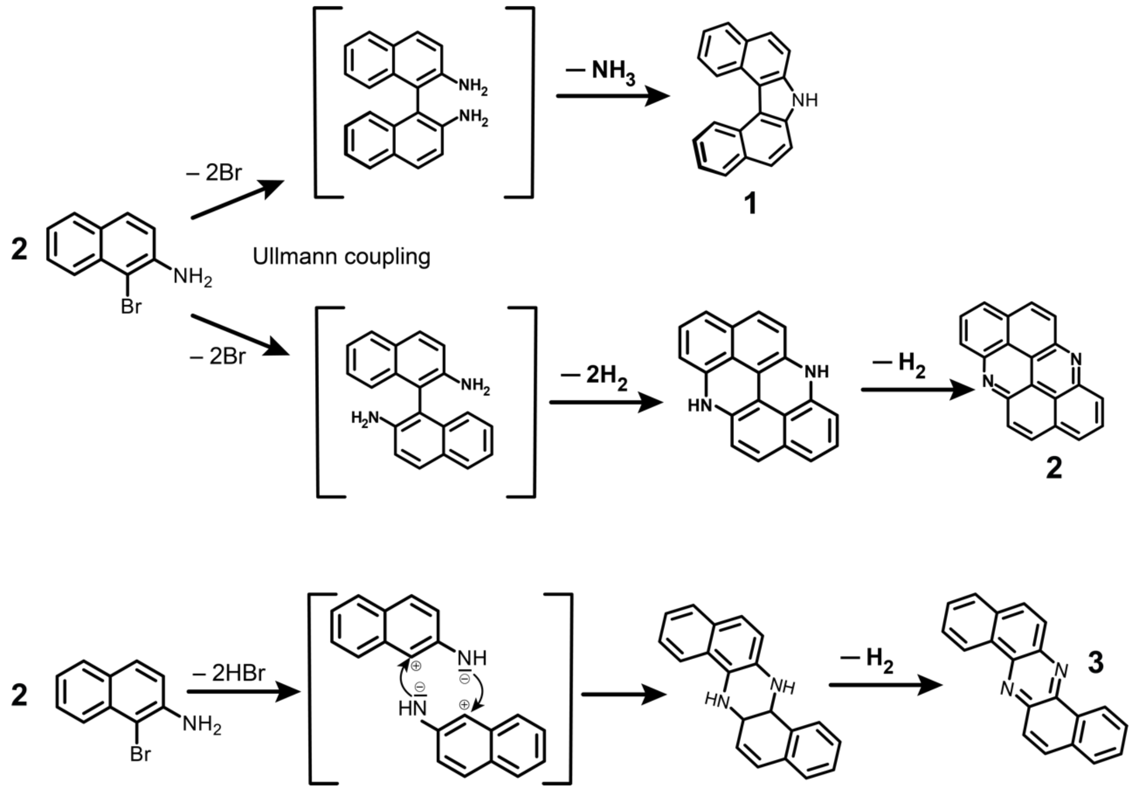

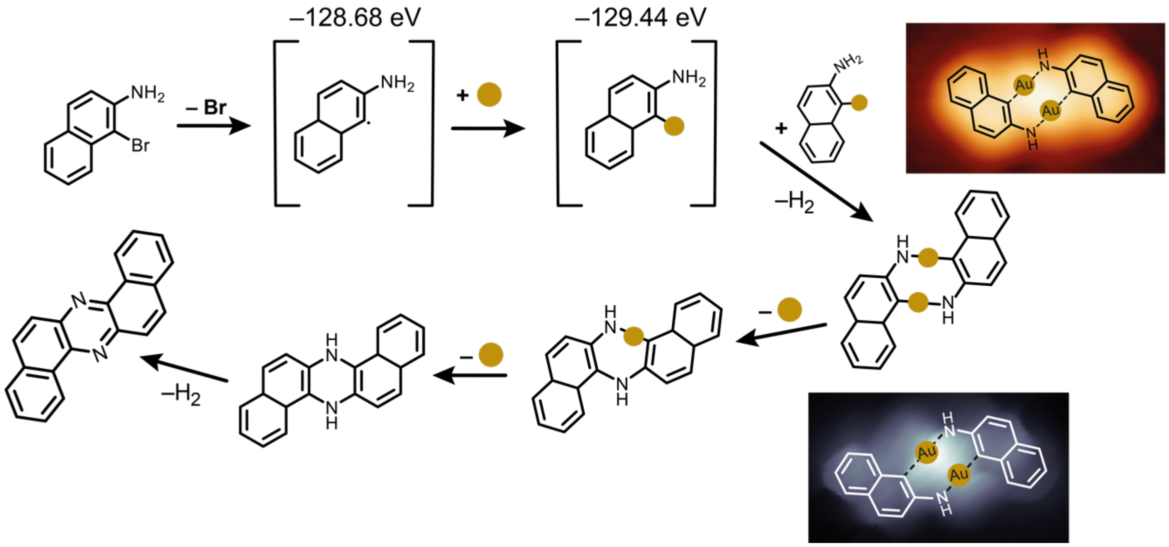

Figure S2. Reaction mechanism under involvement of gold ad-atoms. Energy values for the calculated intermediates on the surface before dimerization are indicated. The Au-passivated radical as intermediate is lower in energy by 0.76 eV. The large distance between dimers observed in STM after annealing to 433 K indicate the presence of ad-atoms. After dehydrogenation, molecular hydrogen desorbs from the surface.

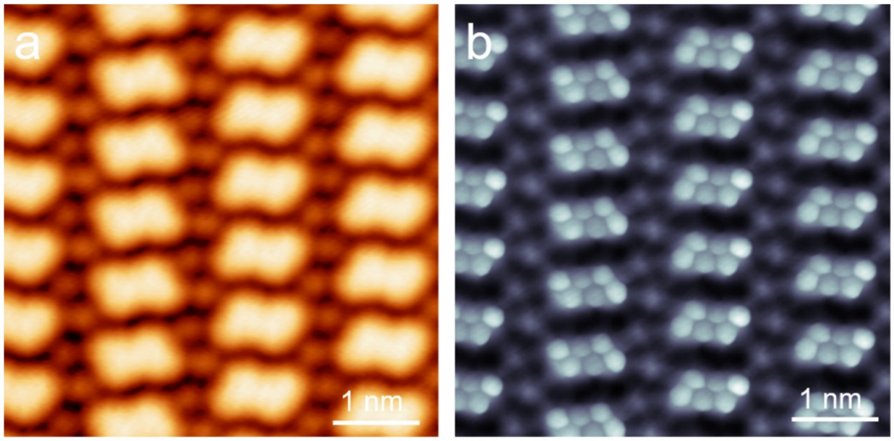

Figure S3. STM and BR-STM Images showing bromine assisted self-assembly of 6,12-diazaanthanthrene. The shown domain contains a row of opposite handedness. Scanning parameters: (a) U = 50 mV, I = 120 pA; (b) U = 2 mV, I = 50 pA.

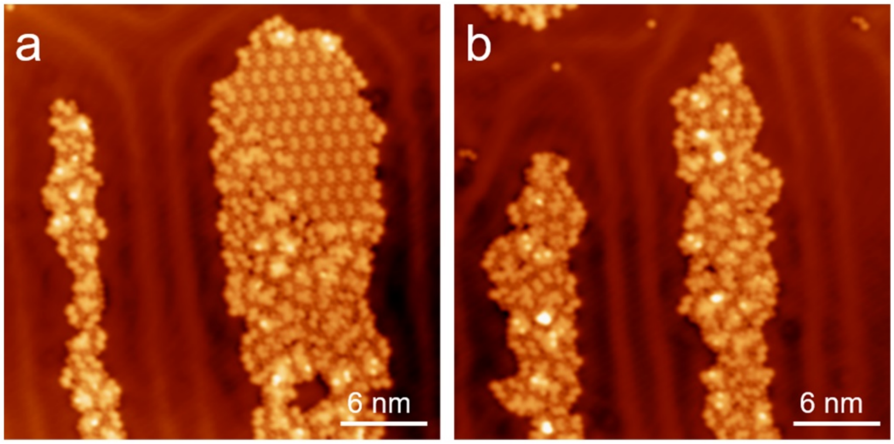

Figure S4. STM images containing ordered and disordered regions. Scanning parameters: (a,b) U = –200 mV, I = 120 pA.

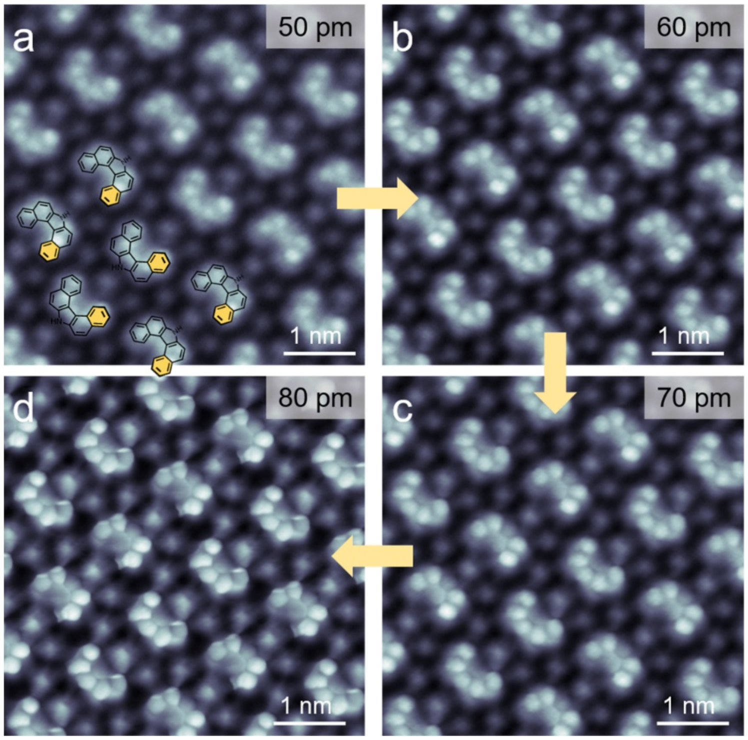

Figure S5. Constant-height BR-STM images at different heights above the surface. (a) 50 pm, (b) 60 pm, (c) 70 pm, (d) 80 pm. Only at low height of the CO-tip above the surface, both enantiomers are clearly distinguishable. Scanning parameters: (a-d) U = 2 mV, I = 50 pA.

1,1’-binaphthyl-2,2’-imine, **1**

	Heterochiral (with Br)	Homochiral (with Br)	Heterochiral (without Br)	Homochiral (without Br)
	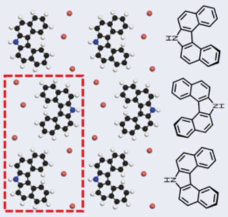	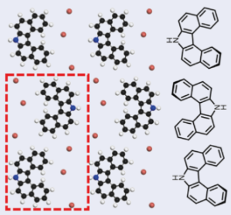	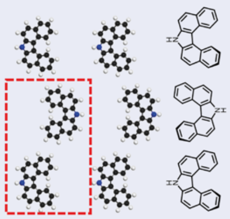	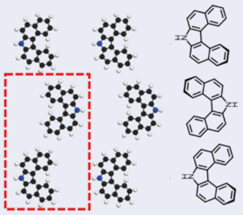
E_B	–5.29 eV per unit cell	–5.27 eV per unit cell	–4.97 eV per unit cell	–4.94 eV per unit cell

dibenzo[a,h]phenanzine, **3**

	Homochiral (with Br)	Heterochiral (with Br)	Homochiral (without Br)	Heterochiral (without Br)
	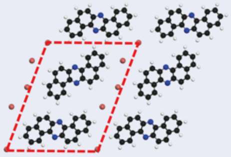	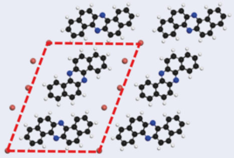	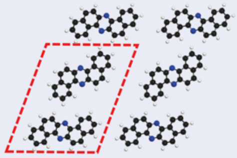	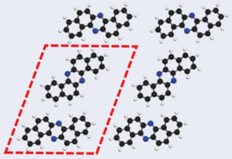
E_B	–5.18 eV per unit cell	–5.08 eV per unit cell	–4.75 eV per unit cell	–4.72 eV per unit cell

Figure S6. DFT-calculated- binding energies per unit cell-for homochiral and-heterochiral assemblies of product **1** and **3**, evaluated with-and without bromine atoms. When- bromine atoms are present, they are also included in the molecule-free reference surface; the reported energies therefore reflect only the molecule-gold interaction. Binding energies are substantially larger in- the presence of bromine, consistent with-the experimental-observations. With respect to chiral self-assembly, the calculations also agree with experiment; however, the energy differences are small and should not be regarded as definitive-confirmation. The binding energy (E_b) is defined as $E_b = E_{tot} – E_{mol} – E_{sub}$, where E_{tot}, E_{mol}, and E_{sub} represent the energies of the total adsorbed system, the molecular assembly in its adsorbed geometry, and the clean Au(111) substrate, respectively.
